# Ring-Opening Polymerization of 1,3-Benzoxazines via Borane Catalyst

**DOI:** 10.3390/polym10030239

**Published:** 2018-02-27

**Authors:** Mustafa Arslan, Baris Kiskan, Yusuf Yagci

**Affiliations:** Department of Chemistry, Istanbul Technical University, Maslak, Istanbul 34469, Turkey; mustafaarslan32@gmail.com

**Keywords:** ring-opening polymerization, benzoxazines, polybenzoxazines, curing temperature, tris(pentafluorophenyl)borane

## Abstract

Tris(pentafluorophenyl)borane was used as Lewis acid catalyst to lower the ring opening polymerization temperature (ROP) of 1,3-benzoxazines. Dynamic scanning calorimeter studies revealed that on-set ROP temperatures were decreased as much as 98 °C for model benzoxazine compounds. Catalytic polymerization was traced by both FTIR and ^1^H NMR, and revealed that tris(pentafluorophenyl)borane acted rapidly and fast curing achieved. Moreover, thermal properties of resulting polybenzoxazines were investigated by thermogravimetric analysis (TGA) and found out that the catalyst has high impact on char yield and even 3 mol % catalyst augmented char yields up to 13%.

## 1. Introduction

**Scheme 1:**
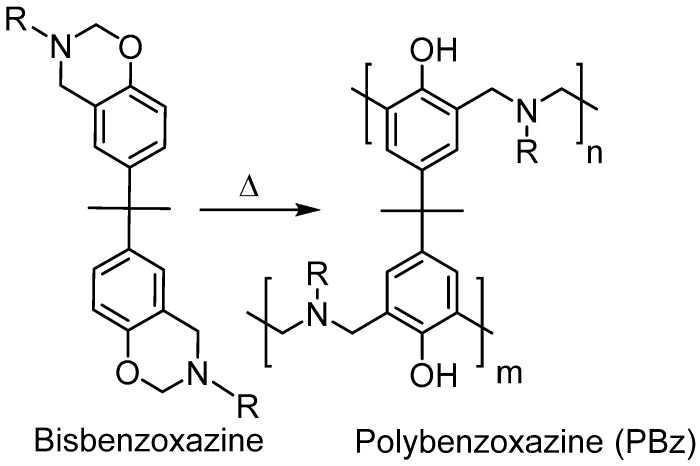
Ring opening polymerization (ROP) of a bisbenzoxazine monomer.

Polybenzoxazines have attracted interest in the last decade due to superior properties in the field of high performance materials. These polymers can be considered as a class of phenolic thermosets resembling to novolac and resole type systems. Thus, they bear in many properties of traditional phenolics, such as excellent mechanical and thermal properties. Besides, polybenzoxazines exhibit unique features that makes them a contender to many high performance polymers. They have low water absorption, molecular design flexibility, and high char yield, and generally high glass transition temperatures [1,2,3,4,5,6]. Most of these features are mainly caused by the aminoalkyl repeat units (–CH_2_–N(R)–CH_2_–) and hydrogen bonds between amine and phenolic –OH groups [7,8,9]. Moreover, the cyclic structural nature of corresponding 1,3-benzoxazine monomers limit shrinkage during curing and also stabilize the monomer at a wide temperature range from room temperature to *ca*. 150 °C, depending on the functionalities that are present on the monomer. [10] Thus, these monomers can be stored for prolonged times even under humid and relatively hot environment. The stability of oxazine ring, on the other hand, makes the polymerization be conducted at high temperatures that can be considered as a drawback [11]. Generally, polybenzoxazines are synthesized by thermally induced ring opening polymerization (ROP) of 1,3-benzoxazines over 220 °C (Scheme 1). These values are higher than usual curing temperatures (*ca*. 177 °C) that are used to produce composites especially for aero-structures. Therefore, reduction in the cure temperature is highly desirable for industrial applications.

**Scheme 2:**
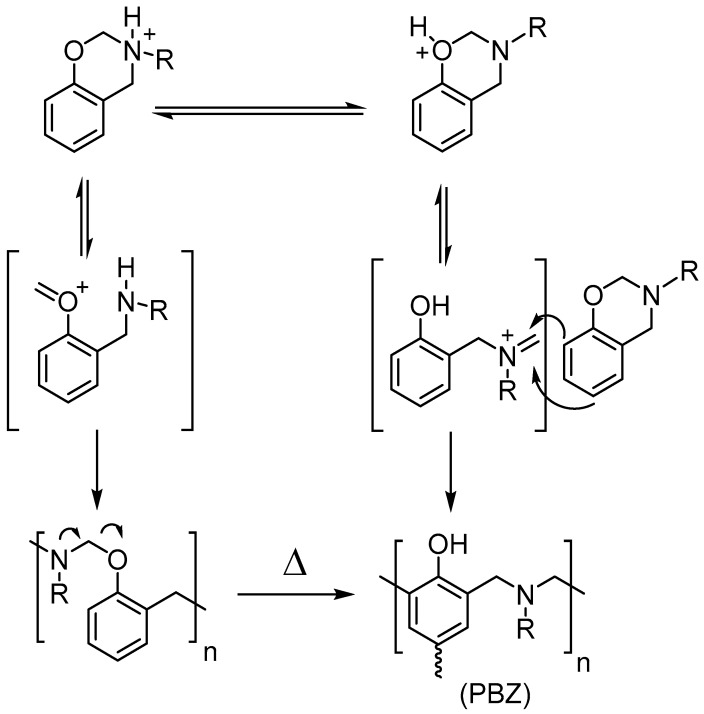
Proposed mechanism for ROP of 1,3-benzoxazines.

The polymerization of benzoxazines proceeds through a cationic ring opening pathway where the oxygen and nitrogen on the heterocycle easily stabilizes the formed cations during the process [12,13,14]. This way, the distorted semi-chair oxazine ring is opened at high temperatures leading to Friedel-Crafts reaction at *ortho* or *para* position of aromatics. Actually, two major mechanistic paths take place according to the cation formation either on nitrogen or oxygen atoms that are on the oxazine ring. If the cation is formed on the oxygen atom of oxazine, then polybenzoxazine is thoroughly produced over ring-opening and Friedel-Crafts reactions. However, cation formation on the nitrogen atom causes ring-opening and subsequent etherification to give –OH blocked polybenzoxazine that eventually rearranges to form phenolic polybenzoxazine at high temperatures (Scheme 2) [15,16].

The nature of polymerization of 1,3-benzoxazines is suitable to be catalysed by acids or other species that are able to produce cation on the oxazine ring. Designing self-catalytic monomers and admixing catalysts with benzoxazines are the usually applied strategies to reduce the curing temperature. Both approach has superiorities and drawbacks, thus selection may vary according to the specific application. For the first strategy, several different benzoxazine monomers containing –COOH and –OH groups were reported [17,18,19,20,21]. In this connection, it should be pointed out that although acidic groups readily reduce the ROP temperatures, decarboxylation observed during the polymerization causes voids in the final polymers due to released CO_2_ is the major deterrent of this strategy [22]. The materials formed this way may exhibit drastic mechanical failures in most applications. Therefore, carboxylic acid containing benzoxazines were best suited to produce sponge like networks, typically for chromatographic column applications. Alternative approach involving the use of –OH functional benzoxazines as self-catalysts appears to be more beneficial since problems associated with gas release is prevented [23]. The ROP temperature reductions were around 40 °C as the interaction of –OH groups with the cationic intermediates formed during ring opening process accelerates the polymerization. Notably, monomers with unoccupied phenolic –OH groups act as built-in catalysts and effectively reduce ROP temperatures which can be considered as an alternative solution to harsh curing conditions [24]. Actually, the described self-catalyst strategy is designed for the systems that contain only benzoxazines and derivatives. On the other hand, this approach needs synthesis of structurally related monomers and for some monomers multi step synthetic routes may be required. As stated, the second strategy, based on addition of catalysts into benzoxazine formulations, appears to be easier and more flexible. Several compounds, namely amines, amine salts, thiols, phenols, toluene sulfonates, and colloidal sulfur were used to promote ROP of benzoxazines [25,26,27,28,29,30,31,32,33]. Moreover, admixing Lewis acids with benzoxazines profoundly affected the curing and ROP temperatures were drastically reduced down to 120–160 °C. Typically, PCl_5_, POCl_3_, TiCl_4_, AlCl_3_, and FeCl_3_ acted as efficient catalysts, particularly when with solvents and ring opening of oxazine took place even at room temperature [34,35,36]. Although these catalysts are economical and potent, their miscibility in neat benzoxazines is limited. Therefore, Lewis acids in complex structures would obviously increase both their solubility and stabilities. Acetylacetonato complexes of 4th period transition metals can be given as a striking example in this context. These complexes with nucleophiles promote the polymerization efficiently and 120 °C as onset of ROP temperatures were obtained [37]. Highly active catalytic nature of Lewis acid often initiate ROP at low temperatures and viscosity increases rapidly, providing a practical advantage for fast curing requirements. Thus, miscibility of the Lewis catalyst come into prominence for such purposes and tris(pentafluorophenyl)borane (B(C_6_F_5_)_3_) has a potential in this manner. It was generally used to catalyse several organic reactions and it also played role in borylation chemistry [38]. For example, substituted quinolones were synthesized by B(C_6_F_5_)_3_ initiated aldehyde-aniline-alkyne reaction [39]. Moreover, B(C_6_F_5_)_3_ was shown to be an excellent activator component in homogeneous Ziegler-Natta processes [40]. Besides its catalytic activity, this acid was used to prepare carbonyl-borane adducts showing solid state luminescence [41] and also as receptor to detect NH_3_ on an organic field-effect transistor [42]. The multirole of B(C_6_F_5_)_3_ prompted us to further extend its usage in other areas particularly in benzoxazine based thermosets. Herein, we present our investigations for the impact of the B(C_6_F_5_)_3_ on ROP of benzoxazines.

## 2. Materials and Methods

### 2.1. Materials

4,4′-Isopropylidenediphenol (bisphenol A) (Aldrich, 97%, St. Louis, MO, USA), aniline (Aldrich, ≥99.5%, St. Louis, MO, USA) paraformaldehyde (Acros, 96%, Geel, Belgium), benzylamine (Aldrich, 99%), 4-*tert*-butylphenol (Aldrich, 99%, St. Louis, MO, USA), butylamine (Aldrich, ≥99%, St. Louis, MO, USA), xylenes (Aldrich, ≥96%, St. Louis, MO, USA), ethanol (Aldrich, ≥99.5%, St. Louis, MO, USA), toluene (Carlo Erba, 99.5%, Barcelona, Spain), tetrahydrofuran (Sigma-Aldrich, ≥99%, St. Louis, MO, USA), hexane (Aldrich, 95%, St. Louis, MO, USA), and diethyl ether (Aldrich, ≥98%, St. Louis, MO, USA), sodium hydroxide (Sigma-Aldrich, ≥98%, St. Louis, MO, USA) were used as received.

### 2.2. Characterization

All ^1^H NMR spectra were recorded on an Agilent NMR System VNMRS 500 spectrometer (Agilent, Santa Clara, CA, USA) at room temperature in CDCl_3_ with Si(CH_3_)_4_ as an internal standard. FT-IR spectra were recorded on a Perkin-Elmer FT-IR Spectrum (Boston, MA, USA) One spectrometer. Differential Scanning Calorimetry (DSC) was performed on Perkin–Elmer Diamond DSC from 0 to 320 °C, with a heating rate of 10 °C/min. under nitrogen flow. Thermal gravimetric analysis (TGA) was performed on Perkin-Elmer Diamond TA/TGA with a heating rate of 10 °C/min under nitrogen flow. Gel permeation chromatography (GPC) measurements were performed on a Viscotek GPC max auto sampler system (Malvern, UK) consisting of a pump, a Viscotek UV detector, and Viscotek a differential refractive index (RI) detector with three ViscoGEL GPC columns (G2000H HR, G3000H HR, and G4000H HR, 7.8 mm internal diameter, 300 mm length) in series. Tetrahydrofuran (THF) was used as an eluent at flow rate of 1.0 mL·min^−1^ at 30 °C. Both of the detectors were calibrated with PS standards having narrow-molecular-weight distribution. Data were analysed using ViscotekOmniSEC Omni-01 software (Malvern, UK).

### 2.3. Synthesis of Aniline Based Bisbenzoxazine (B-a)

In a 100 mL round bottomed flask, paraformaldehyde (34.9 mmol, 1.05 g), bisphenol A (8.76 mmol, 2.0 g), and aniline (17.5 mmol, 1.6 mL) were dissolved with 20 mL of xylenes. The reaction mixture was heated to 120 °C for 12 h. The solvent was evaporated under vacuum. The resulting oily product was dissolved in diethyl ether (200 mL) and extracted with 3 M sodium hydroxide for three times to remove unreacted bisphenol A. Then, to neutralize the solution diethyl ether solution washed with distilled water (150 mL) for three times. The solution was dried with anhydrous Na_2_SO_4_ and filtered. Diethyl ether was evaporated under vacuum. The product was dried under vacuum at room temperature for 24 h.

### 2.4. Synthesis of Benzylamine Based Monobenzoxazine (P-Bn)

In a 100 mL round bottomed flask, paraformaldehyde (106 mmol, 3.19 g), phenol (53.1 mmol, 5.0 g), and benzylamine (53.1 mmol, 5.80 mL) were dissolved with 50 mL of toluene and 25 mL of ethanol mixture. The reaction mixture was refluxed for 12 h. The solvent was evaporated under vacuum. The resulting oily product was dissolved in diethyl ether (200 mL) and extracted with 3 M sodium hydroxide for various times to remove unreacted bisphenol A. Then, to neutralize the solution diethyl ether solution washed with distilled water (150 mL) for three times. The solution was dried with anhydrous Na_2_SO_4_ and filtered. Diethyl ether was evaporated under vacuum. The product was dried under vacuum at room temperature for 24 h.

### 2.5. Synthesis of 4-t-butylphenol Based Monobenzoxazine (t-P-Bt)

4-*t*-Butylphenol (5.0 g, 33.3 mmol) was added to a 100 mL round bottomed flask and cooled to 0 °C in an ice-bath. Butylamine (2.43 g, 33.3 mmol) was added dropwise to 4-*tert*-butylphenol for 15 min. and stirred for 5 min. Paraformaldehyde (1.99 g, 66.4 mmol) was added partly to the mixture over 20 min with stirring at 0 °C. After the mixture was stirred over 15 min. at room temperature, temperature was raised to 105 °C and stirred for 2 h. The reaction medium cooled to room temperature, dissolved in diethyl ether and extracted with aqueous sodium hydroxide (10 wt %, 250 mL), two times with distilled water and dried with magnesium sulfate. The product was dried under vacuum at room temperature for 24 h.

## 3. Results and Discussion

**Scheme 3:**
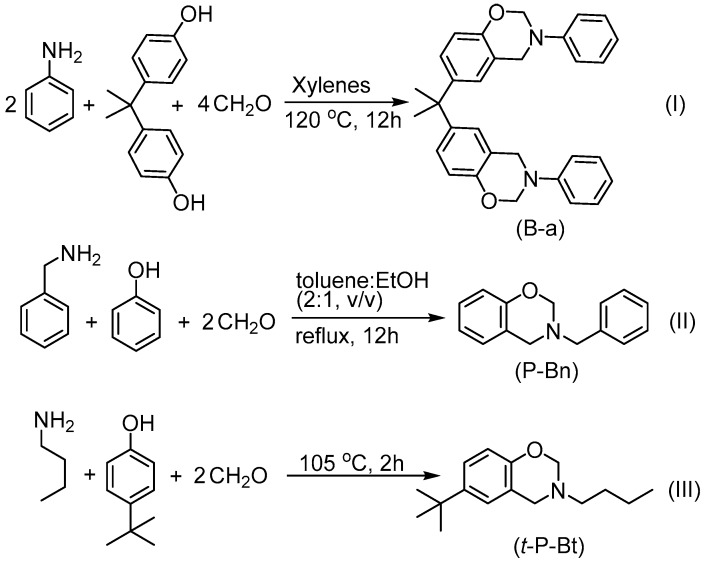
Synthesis of B-a, P-Bn, *t*-P-Bt monomers.

As stated, previous studies on the ROP mechanism of benzoxazines disclosed that the polymerization is initiated by thermally induced formation of cationic intermediates on N– and O– atoms, followed by the concurrent cleavage of methylene bridge on the oxazine ring. The formed cationic species immediately attack N–, O–, or aryl group and the rearrangement of labile bonds finally produce polybenzoxazines. In such a polymerization mechanism, Lewis acids are appropriate to consider as effective catalyst system since Lewis acids can strongly bind and generate cationic species on nitrogen and oxygen atoms of oxazine ring. Thus, the energy requirement for cleaving the oxazine ring reduces drastically and the ROP temperature decrease becomes imminent. It should be noted that the solubility of many Lewis acids are limited in organic compounds, and thus, in a benzoxazine medium, the solubility of the acid and polarity of benzoxazine monomer would affect the catalyst performance. Therefore, preparation of benzoxazine based formulations containing Lewis acids, such as FeCl_3_, TiCl_4_, AlCl_3_, ZnCl_2_ can be problematic due to their low solubility in organic solvents. Alternatively, ligated Lewis acids may provide better solubility and catalytic activity. Apart from solubility issues, boron compounds are known to show high complexation affinity against amines and oxazine ring bearing tertiary amine in its structure, which would presumably strongly bind central boron atom of a boron based Lewis acid [43]. It seemed, therefore, appropriate to investigate the catalytic activity of B(C_6_F_5_)_3_ on the ROP of benzoxazines. Initially, mono and bisbenzoxazines were synthesized as model monomers by using conventional method, according to the literature (Scheme 3) [44,45,46]. Spectral characterization of these monomers was performed using ^1^H NMR analysis (Figures S1–S3).

**Figure 1:**
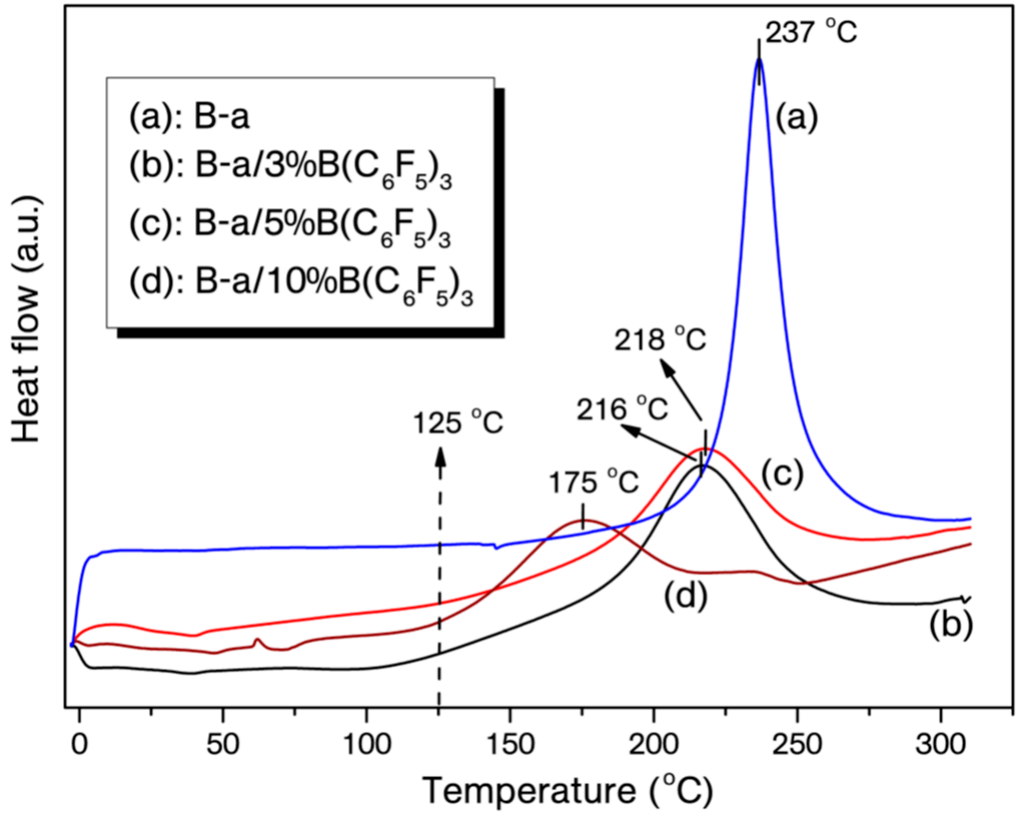
Differential Scanning Calorimetry (DSC) thermograms of pristine B-a (**a**), B-a/3%B(C_6_F_5_)_3_ (**b**), B-a/5%B(C_6_F_5_)_3_ (**c**), B-a/10%B(C_6_F_5_)_3_ (**d**).

**Figure 2:**
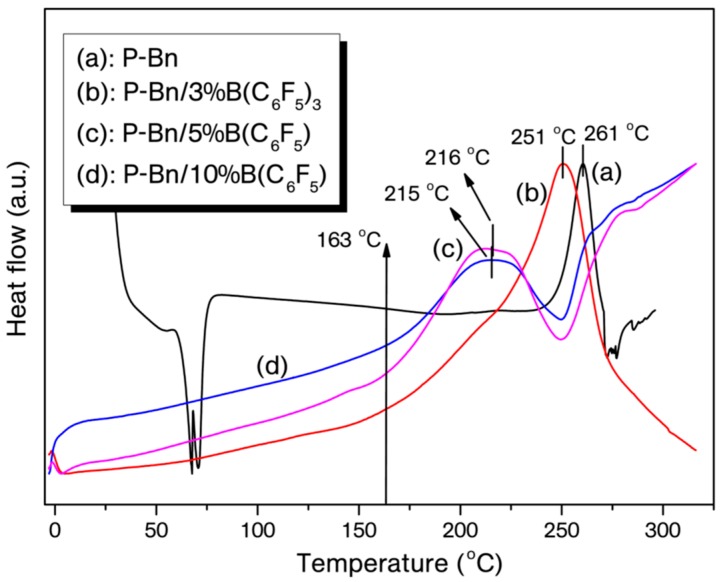
DSC thermograms of pristine P-Bn (**a**), P-Bn/3%B(C_6_F_5_)_3_ (**b**), P-Bn/5%B(C_6_F_5_)_3_ (**c**), P-Bn/10%B(C_6_F_5_)_3_ (**d**).

Typically, the catalyst was admixed with benzoxazine monomers in either 3 or 5 mol % by dissolving the components in tetrahydrofuran (1 mL) to obtain homogeneous mixtures. The solvent in the mixtures were removed by blowing nitrogen gas and then subjected to vacuum (*ca*. 10 mm-Hg) for 5 min. at room temperature. As is known, most of the benzoxazines have the ROP temperatures (curing maximum, *T*_max_) generally over 220 °C, which can be monitored as a distinct exotherm. Thus, ROP of B-a and P-Bn with B(C_6_F_5_)_3_ were traced under N_2_ environment with a heating rate of 10 °C·min^−1^ using a DSC device. The overlaid DSC thermograms for benzoxazine/catalyst mixtures presented in Figure 1 and Figure 2 and DSC data tabulated in Table 1 clearly show the ROP temperature reduction of these monomers with added B(C_6_F_5_)_3_. Moreover, the impact of catalyst amount on the ROP was also examined by increasing the B(C_6_F_5_)_3_ content from 3 to 10 mol % and the on-set of curing temperatures dropped to 125 from 212 °C for B-a and 163 from 242 °C for P-Bn (Figure 1 and Figure 2), corresponding to 98 and 81 °C reduction on on-set curing temperature, respectively.

**Scheme 4:**
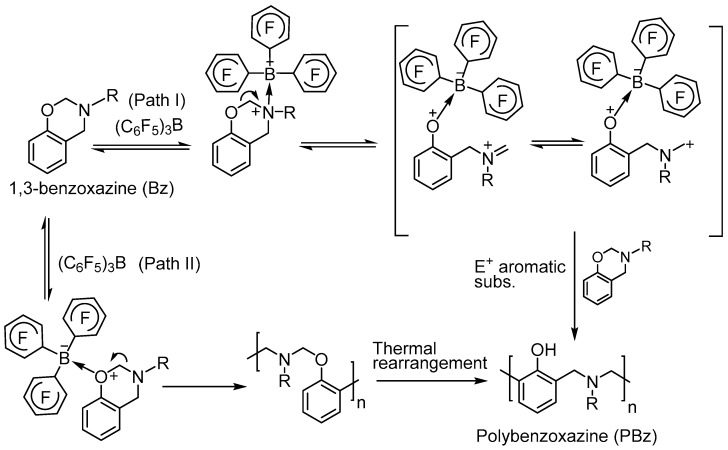
Tris(pentafluorophenyl)borane catalysed ROP of benzoxazine.

Previous mechanistic studies concerning Lewis acid catalysed ROP of benzoxazines disclosed the characteristic and two major polymerization mechanism was proposed. Correspondingly, a plausible mechanism for the B(C_6_F_5_)_3_ promoted ROP is proposed in Scheme 4. In the case of boron compounds, path I appears to be dominant process when compared to path II, due to the high complexation affinity of N and B atoms thus favours the electron transfer from N to B in the first step.

**Figure 3:**
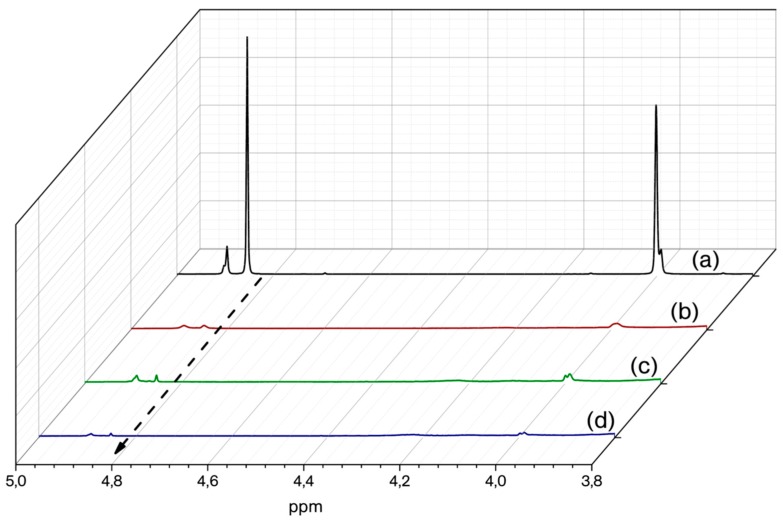
Overlaid ^1^H NMR spectra of *t*-P-Bt/5% B(C_6_F_5_)_3_ mixture as prepared (**a**); *t*-P-Bt/5% B(C_6_F_5_)_3_ mixture after 30 min (**b**); *t*-P-Bt/5% B(C_6_F_5_)_3_ mixture after 60 min (**c**); *t*-P-Bt/5% B(C_6_F_5_)_3_ mixture after 120 min (**d**).

It was shown that linear and soluble polybenzoxazines can be obtained by using mono functional benzoxazines with blocked para position on the phenol moiety. For example, blocking the para position with tertiary butyl groups prevents the attack of carbocation to this position thus avoids crosslinking. Hence, *t*-P-Bt was deliberately selected so as to follow ring-opening reaction by ^1^H NMR. Initially, the solubility of the cured *t*-P-Bt was tested after curing at 220 °C for 1 h and the obtained polymer was analysed by GPC using THF as eluent to determine the molecular weight. The GPC chromatogram (Figure S4) showed a molecular weight of *M*_n_: 980 Da with 3.01 polydispersity index. The relatively high dispersity observed is the consequence of the nature of the condensation polymerizations. As stated, the solubility of B(C_6_F_5_)_3_ in the reaction medium is a crucial factor for the ring-opening of benzoxazines. It is even likely that ring-opening can be achieved at r.t. in certain solvents. However, it is important to point out that it does not necessarily mean that the overall polymerization can take place at r.t. To test this probability, we have conducted a reaction followed by ^1^H NMR spectroscopy. ^1^H NMR spectra of the aliquot samples of the solution of *t*-P-Bt and B(C_6_F_5_)_3_ (5 mol %) in CDCl_3_ at the beginning and after 30, 60, and 120 min were recorded to observe the changes of the signals corresponding to N–CH_2_–O protons of the oxazine ring. As can be seen from Figure 3, the intensity of the peak at 4.85 ppm decreases drastically within 30 min., indicating that B(C_6_F_5_)_3_ catalyses ring-opening reaction at r.t. in solution medium. These results reveal that the catalyst acts rapidly and starts the ring-opening upon admixing, thus provide clear evidence for the fast curing at higher temperatures.

**Figure 4:**
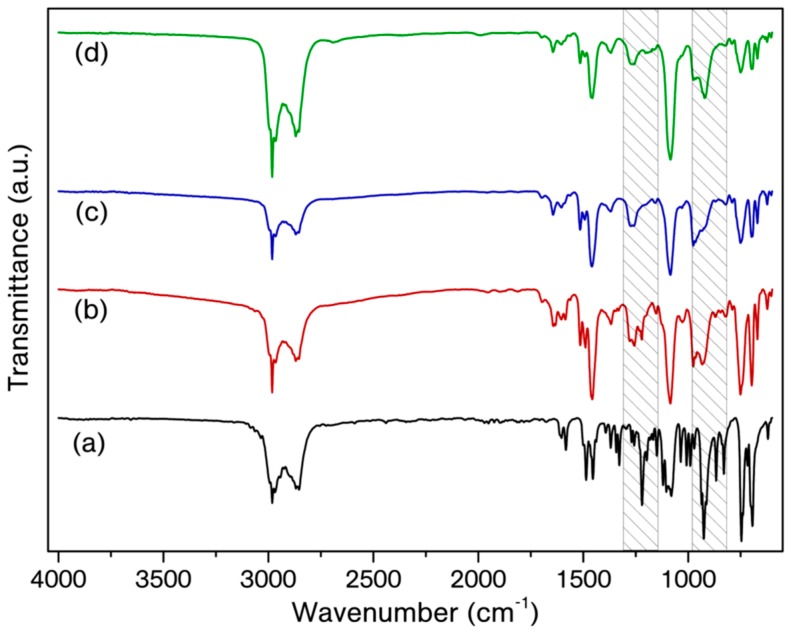
Overlaid FTIR spectra of P-Bn/5% B(C_6_F_5_)_3_ mixture as prepared (**a**), P-Bn/5% B(C_6_F_5_)_3_ mixture after 30 min. (**b**), P-Bn/5% B(C_6_F_5_)_3_ mixture after 60 min. (**c**), P-Bn/5% B(C_6_F_5_)_3_ mixture after 120 min. (**d**).

On the other hand, a similar curing study was also performed by using P-Bn monomer, which forms crosslinked polybenzoxazine. Therefore, FTIR spectroscopy was used to track the ring-opening of oxazine ring for certain time intervals (Figure 4). The overlaid FTIR spectra of the resulting products clearly reveals the change on the oxazine ring bands. The intensity of the C–O–C band at 1218 cm^−1^ is reduced after 30 min and the reduction becomes more pronounced after 60 min. Moreover, polymerization of benzoxazine resins is generally proved by the disappearance of the band at 960–900 cm^−1^, corresponding to the C–H out-of-plane bending of the benzene and the O–C stretching of the oxazine ring [47]. The intensity of the related band at 926 cm^−1^ is reduced by time and a new band at 967 cm^−1^ emerges, evidencing the ring-opening of oxazine by B(C_6_F_5_)_3_ catalyst.

**Figure 5:**
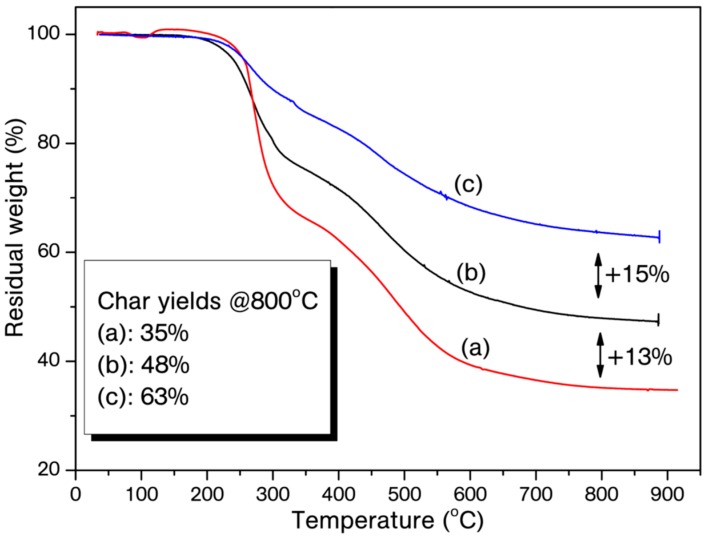
Thermo-gravimetric analysis (TGA) traces of cured P-Bn (**a**), P-Bn/3% B(C_6_F_5_)_3_ (**b**), P-Bn/5% B(C_6_F_5_)_3_ (**c**).

**Figure 6:**
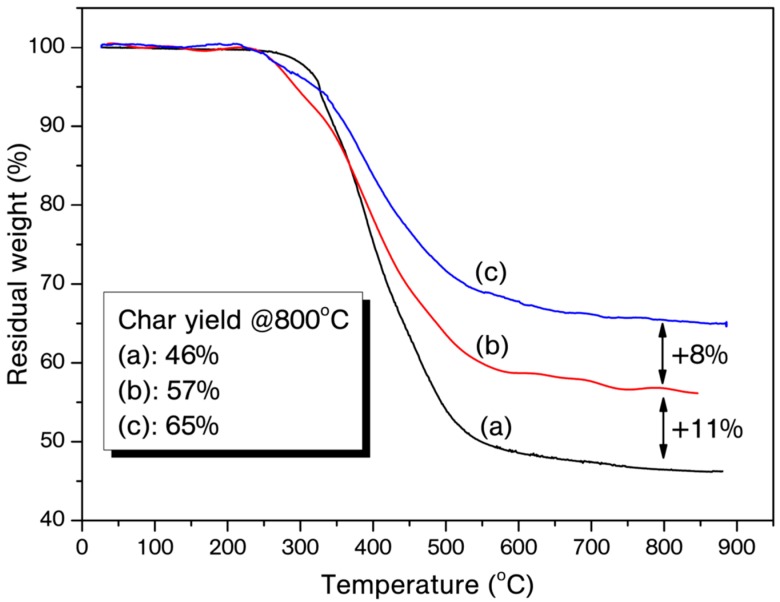
TGA traces of cured B-a (**a**), B-a/3% B(C_6_F_5_)_3_ (**b**), B-a/5% B(C_6_F_5_)_3_ (**c**).

The thermal stability of the polybenzoxazines for each catalyst ratio was studied by using thermo-gravimetric analysis (TGA). TGA traces and related thermo-gravimetric results are presented in Figure 5 and Figure 6, and Table 2, respectively. Although cured P-Bn/B(C_6_F_5_)_3_ have slight differences for T_5%_, a pronounced effect of the catalyst on the thermal stability for *T*_10%_, *T*_c_ and *T*_max_ values is visible. On the other hand, the initial degradation values for cured B-a/B(C_6_F_5_)_3_ are lower than that of the cured pristine B-a. Polybenzoxazines formed from monofunctional benzoxazines generally have low crosslinking degree and the catalyst presumably increases the crosslinking density of polybenzoxazine of P-Bn. In contrast, B-a already produces stiff network due to the difunctional structure, thus any positive effect on initial thermal degradation is not observed. Notably, the addition of B(C_6_F_5_)_3_ showed drastic increment in the char yield at 800 °C for both of the systems. Similar positive effect was also observed for polybenzoxazines obtained by transition metal based catalytic ROP. Although the B atom is not a transition metal, char yield improvement is significant with B(C_6_F_5_)_3_ catalyst and only 3 mol % addition of B(C_6_F_5_)_3_ augmented the char yields 11–13%. Three reasons can be discussed to explain this phenomenon. The high degree of crosslinking due to the catalyst, the coordination between boron and nitrogen atoms that may delay the amine degradation in polybenzoxaine, and apart from central boron atom in the catalyst, fluorine atoms may also contribute to this observed effect due to the well-known flame retardation property of aromatic fluorine compounds [48,49].

## 4. Conclusions

We have established the catalytic potential of tris(pentafluorophenyl)borane in the polymerization of benzoxazines. The benzoxazine/catalyst mixtures were easily prepared and the catalyst solubility was high due to the its ligated nature and the strong binding affinity between N and B atom. As expected, borane catalyst acted rapidly and initiated the ROP of model benzoxazine compounds, which disclosed that the catalyst is suitable for fast curing demands. The strong catalytic properties of the catalyst exhibited an unusual impact on thermal properties of the resulting polybenzoxazines. Especially, char yield improvement is significant with the catalyst and only 3 mol% addition of B(C_6_F_5_)_3_ augmented the char yields 11–13%. Therefore, this catalyst may find applications where high char yields are required and metal based catalyst impurities would be a drawback.

## Figures and Tables

**Table 1:** DSC ^a^ characteristics of B-a, P-Bn and their mixtures with various amounts of B(C_6_F_5_)_3_.

Entry	On-Set of Curing (°C)	End-Set of Curing (°C)	Maximum Curing (°C)	Δ*H* (J/g)
B-a	212	266	237	−201
B-a/3% B(C_6_F_5_)_3_	180	251	216	−156
B-a/5% B(C_6_F_5_)_3_	180	252	217	−117
B-a/10% B(C_6_F_5_)_3_	125	216	174	−103
P-Bn	242	272	261	−35
P-Bn/3% B(C_6_F_5_)_3_	217	270	251	−130
P-Bn/5% B(C_6_F_5_)_3_	174	248	216	−45
P-Bn/10% B(C_6_F_5_)_3_	163	247	215	−74

^a^ DSC thermograms were collected under 20 mL·min^−1^ N_2_ flow and 10 °C·min^−1^ heating rate.

**Table 2:** Thermal properties of the cured P-Bn, B-a and B(C_6_F_5_)_3_ (3 and 5 mol %) mixtures.

Sample	*T*_5%_ (°C)	*T*_10%_ (°C)	*T*_max_ (°C) ^a^	*T*_c_ (%)
P-Bn	257	267	270 *, 466	35
P-Bn/3% B(C_6_F_5_)_3_	258	263	272 *, 492	48
P-Bn/5% B(C_6_F_5_)_3_	244	297	268 *, 557	63
B-a	326	364	390	46
B-a/3% B(C_6_F_5_)_3_	294	347	284 *, 392	57
B-a/5% B(C_6_F_5_)_3_	323	340	271 *, 391	65

^a^ These values extracted from derivative of TGA (Figures S5 and S6 in Supplementary Materials); *Major *T*_max_; *T*_5%_: The temperature for which the weight loss is 5%; *T*_10%_: The temperature for which the weight loss is 10%; *T*_max_: The temperature for maximum weight loss; *T*_c_: The char yield at 800 °C.

## Supplementary Materials

The following are available online at http://www.mdpi.com/2073-4360/10/3/239/s1.
