# Supplementary material for: Ring-Opening Polymerization of 1,3-Benzoxazines via Borane Catalyst
Source: Polymers (Basel). 2018 Feb 27;10(3):239. doi: 10.3390/polym10030239 (PMC6415064; doi:10.3390/polym10030239)
Supplement: Supplementary file 1 [file polymers-10-00239-s001.pdf]

Supplementary Materials

Ring-opening polymerization of 1,3-benzoxazines via borane catalyst

Mustafa Arslan[1], Baris Kiskan*[,1], Yusuf Yagci*[,1]

[1] *Istanbul Technical University, Department of Chemistry, 34469, Maslak, Istanbul, Turkey*

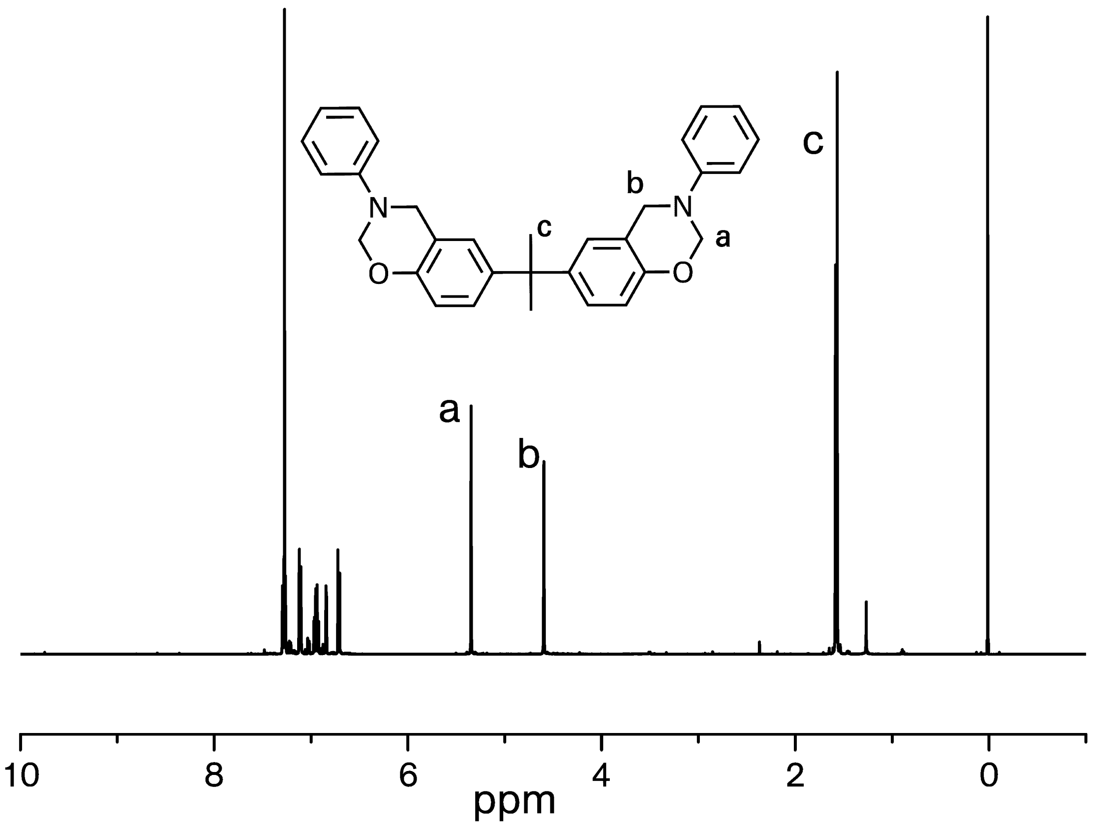

Figure S1: ^{1}H NMR spectrum of B-a monomer

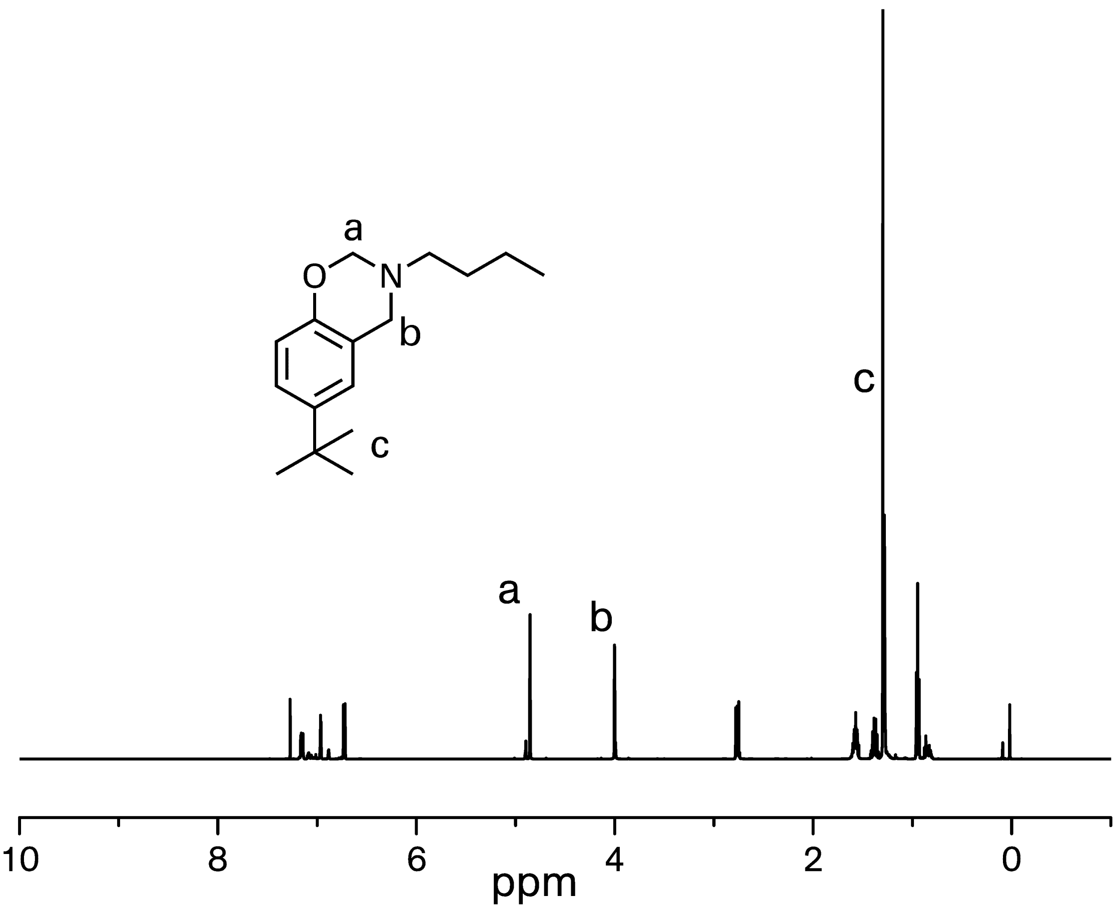

Figure S2: ^{1}H NMR spectrum of *t*-P-Bt monomer

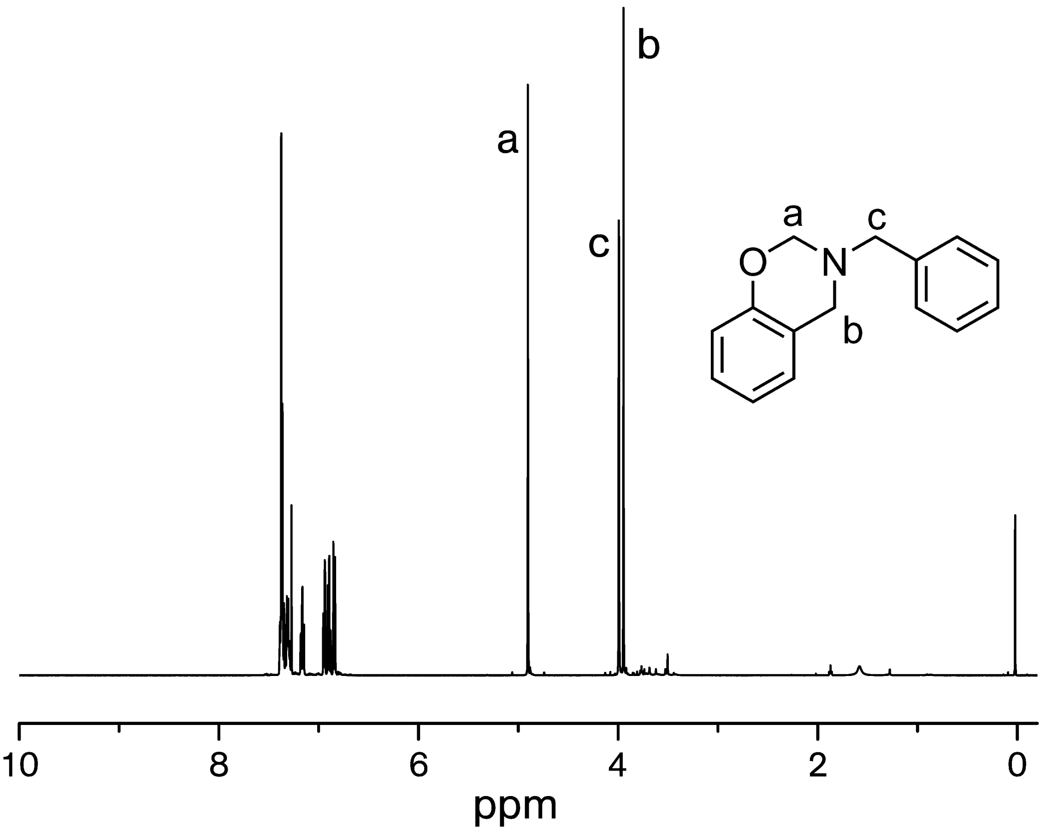

Figure S3: ^{1}H NMR spectrum of P-Bn monomer

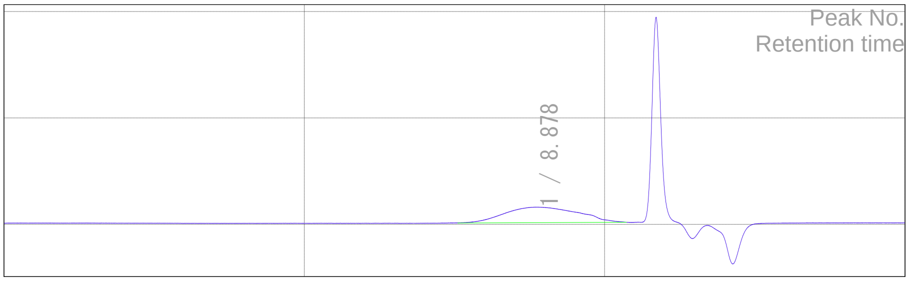

Figure S4: Gel permeation chromatogram of cured *t*-P-Bt

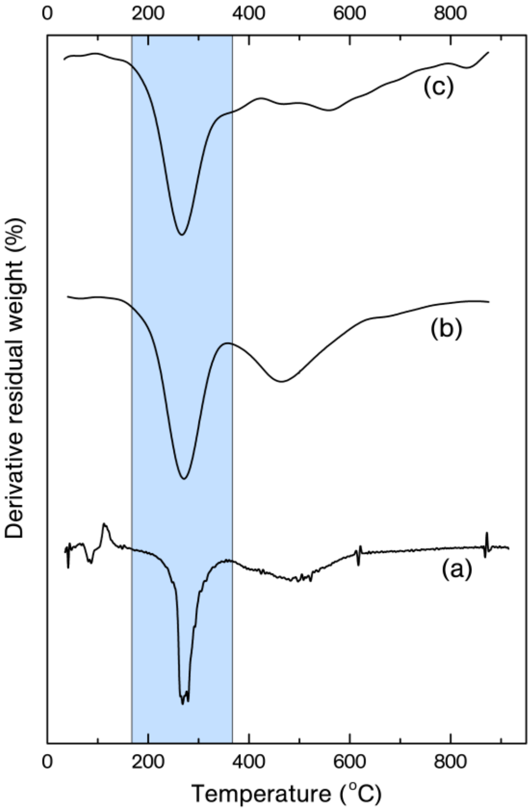

Figure S5: Derivative TGA of cured *t*-P-Bt (b), *t*-P-Bt /3% $B(C_6F_5)_3$ (a), *t*-P-Bt /5% $B(C_6F_5)_3$ (c)

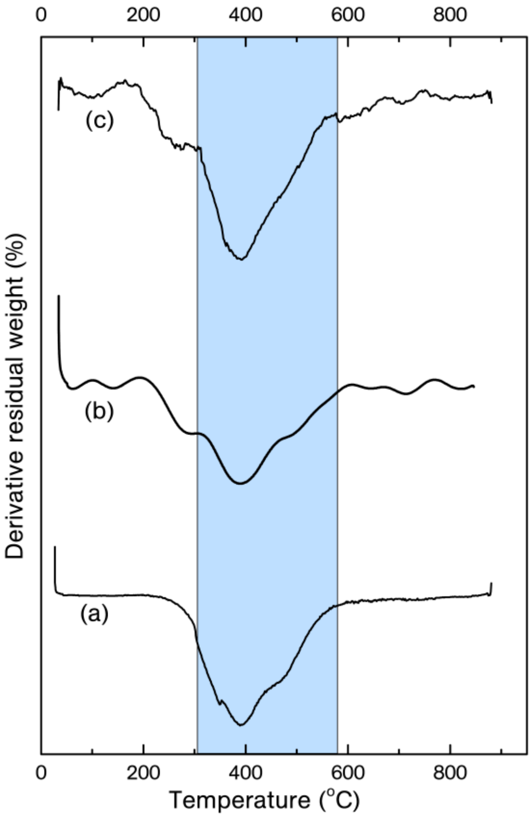

Figure S6: Derivative TGA of cured B-a (a), B-a /3% $B(C_6F_5)_3$ (b), B-a /5% $B(C_6F_5)_3$ (c)
